# Results on a Binding Neuron Model and Their Implications for Modified Hourglass Model for Neuronal Network

**DOI:** 10.1155/2013/374878

**Published:** 2013-12-16

**Authors:** Viswanathan Arunachalam, Raha Akhavan-Tabatabaei, Cristina Lopez

**Affiliations:** ^1^Department of Mathematics, Universidad de los Andes, Bogotá, Colombia; ^2^Department of Statistics, Universidad Nacional de Colombia, Bogotá, Colombia; ^3^Department of Industrial Engineering, Universidad de los Andes, Bogotá, Colombia

## Abstract

The classical models of single neuron like Hodgkin-Huxley point neuron or leaky integrate and fire neuron assume the influence of postsynaptic potentials to last till the neuron fires. Vidybida (2008) in a refreshing departure has proposed models for binding neurons in which the trace of an input is remembered only for a finite fixed period of time after which it is forgotten. The binding neurons conform to the behaviour of real neurons and are applicable in constructing fast recurrent networks for computer modeling. This paper develops explicitly several useful results for a binding neuron like the firing time distribution and other statistical characteristics. We also discuss the applicability of the developed results in constructing a modified hourglass network model in which there are interconnected neurons with excitatory as well as inhibitory inputs. Limited simulation results of the hourglass network are presented.

## 1. Introduction

Several mathematical models of neurons have been developed so that the model neurons mimic biological neurons in various abstract biological features that make these neurons suitable for information processing. In this regard, models of temporal integrator, coincidence detector, and leaky integrate and fire (LIF) of the neuron are computed using the level crossing of the membrane potential. The leak of the membrane potential is at best accommodated using LIF models. However, for the problem of the level crossing of the LIF neuron with instant or curved boundaries, no closed form solution is available and this value can only be computed using numerical methods. Furthermore, the LIF models do not take into account the frequency of the inputs, thereby assuming that the membrane potential integrates the inputs, however large is the interval between them. But it has been observed that during the processing of sensory signals the spiking statistics of individual neurons changes substantially when the signal travels from periphery to more central areas. This aspect lends credence to the point of view of information condensation and supports the theory of finite lifetime of input signals.

Inspired by the findings of numerical simulation of Hodgkin-Huxley [1] neurons as well as LIF models [2], Vidybida [3] proposed models of binding neurons with instantaneous feedback. These are model neurons which mimic real neurons in many biophysical mechanisms. In a binding neuron, any input impulse is stored for a fixed time period *τ* after which it is lost forever. When the number of stored inputs crosses a fixed threshold Σ, the neuron sends a spike while the stored inputs are erased and the neuron starts receiving fresh inputs with a clean state. One obtains the binding neuron with feedback by the immediate feeding of each output impulse to the neuron's input. In this case, the neuron after the spike has one input stored with life time *τ*. Vidybida [3] using lengthy arguments derived the analytical solution of interspike interval distribution and other statistical characteristics in the limited case of Σ = 2 with inputs forming a Poisson process. He argued that the presence of both deterministic and stochastic (Poisson inputs) dynamics in the system leads to fixed *τ*. This approach is to be contrasted with mass service theory [4], where *τ* can be visualised to be random. Furthermore, the Poisson stream of inputs is realized as the sum of all Poisson intensities in the synapses, which leads to a superposed Poisson process with exponential input distributions. The Markovian property of the input signals renders the analysis simple and leads to analytical solutions. However, one could replace the input distribution with any other distribution. For instance, invoking central limit theorem will lead to Gaussian input distributions [5]. Alternatively, the uniform distribution is a potential candidate for the inputs. This paper addresses the above-mentioned points and generalizes Vidybida's model of binding neurons with instantaneous feedback. For the generalized model, we derive explicitly the probability distribution as well as the statistical characteristics of the firing time of the neuron. We proceed in the sequel to outline how to use these results for a neural network composed of interconnected binding neurons. Towards this end, we choose the hourglass model first proposed by Cottrell et al. [6].

Experimental evidence has clearly shown that the analysis of neural networks requires the spike timings of the neurons connected to the network. Thus, the studies of the spiking neuron models and the simulation of the associated networks have gained impetus in the literature [7–10]. Neuronal networks are extremely complex and are randomly interconnected recurrent networks of neurons. These neurons are connected by spike-driven synapses between excitatory neurons and inhibitory neurons [11–16]. The network models can be broadly classified as simple synchronous and complicated asynchronous models. In the synchronous models, the counter is reset simultaneously for every connected neuron. On the other hand, in the asynchronous models, the connected neurons are updated only when they fire. Cottrell [17] proposed an hourglass asynchronous model to describe the neural activity in a network. While the classical single neuron models focused entirely on the membrane potential at any time *t*, the hourglass model associated at each time *t* the expected time to fire. The expected time to fire of any neuron in a network increases due to the firing of the inhibitory connected neuron while the same is shortened by the firing of a connected excitatory neuron. The utility of Cottrell's approach is that it enables us to model the behavior of the network by the states of a time homogenous irreducible and aperiodic Markov chain. These models have subsequently been analyzed, applied to real systems, and extended in several directions [18–22].

Keeping the aforesaid observations in view, we discuss a modified hourglass model for a neural network composed of binding neurons with instantaneous feedback. The outline of the paper is as follows. A model for the probability density function of the interspike intervals for a single binding neuron with instantaneous feedback is proposed in Section 2. Here, we extend Vidybida's binding neurons for the case Σ = 2 for any renewal counting process of the input impulses. The Poisson process arises as a special case of our formulation. Following the mass service theory of Khinchin [4], we generalize *τ* to be a random variable which allows us to get Vidybida's deterministic case of *τ* as a special case. The statistical characteristics of the spike distribution are obtained explicitly. In Section 3, we utilize the results of Section 2 to analyse a neural network composed of binding neurons in which the spiking dynamics of each neuron is represented by an hourglass metaphor. While the description of the behaviour of the network is similar to that of Cottrell et al. [6], the delay parameter *w*_*ij*_ is modeled based on the interconnecting neuron's characteristics. The results of a limited simulation study which may not be conclusive to analyse the activity levels of the neurons in the network are also presented. We present some observations in the last section.

## 2. Binding Neuron with Instantaneous Feedback

### 2.1. Model Description

Traditional neuron models consider a single neuron, which is excited by external stimulations occurring at random. In the classical integrate and fire neuron models, the membrane potential gets excited with successive stimulations and the neuron fires when the potential level crosses a threshold. These models assume, even with the leak of the membrane potential, that successive stimulations contribute to the firing, however wide these stimulations are separated. The frequency of stimulations in the case of a leaky membrane plays a role in the firing rate. However, this aspect is largely ignored in the classical models. In this regard, binding neurons which are model neurons can adequately describe the signal processing in neural systems [3]. In this section, we develop such a neuron model which will be used to develop a modified hourglass model for interconnected neurons in the later section.

Let us consider a single neuron without lateral connections which is excited by stationary external stimulations. These stimulations occur at random and follow a renewal counting process characterized by the interval probability density function *f*(*t*). We wish to observe that the Poisson process input stream available in the literature is a particular case of the present model. The binding neuron is characterised by the following assumptions. Any arriving impulse has a random lifetime *τ* with distribution function *G*(·) during which it is stored but is completely lost after its lifetime. When the number of stored inputs reaches a threshold *K*, the neuron fires sending out a neural spike. Note that under the present assumption, for a neuron to fire, there must be at least *K* impulses in a length of *τ*. For a binding neuron, the number of stored inputs immediately after a firing is reset to zero. However, one obtains a binding neuron with feedback by immediately feeding each output impulse to the neuron's input. In this case, just after firing, the neuron starts with one impulse in its memory which has a life time *τ*. One needs to observe the analogy between the threshold of the membrane potential for level crossing so that the neuron can fire and the threshold of the number of stored inputs *K*. We also wish to observe that the present model maps the sequence of input impulses on to the sequence of neuronal firing outputs determined by the threshold *K*. The two sequences are related by a one-to-one correspondence and may be reconstructed from each other. This leads us to the process of information condensation.

*Notation*

- *Z*: random variable denoting the time between stimuli
- $f_{Z},F_{Z},\bar{F_{Z}}$: pdf, cumulative distribution, and survival functions of *Z*
- *τ*: random variable denoting the lifetime of each stimuli
- $g_{\tau },G_{\tau },\bar{G_{\tau }}$: pdf, cumulative distribution, and survival functions of *τ*
- *K*: threshold of the number of stored inputs for the neuron to fire
- *T*: random variable denoting the time between two consecutive spikes
- $h_{T},H_{T},\bar{H_{T}}$: pdf, cumulative distribution, and survival functions of *T*
- *L*_*f*_(*s*): Laplace transform of the density function *f*(*t*).

### 2.2. The Model

Our proposed model for the firing of a single binding neuron with instantaneous feedback is governed by the following assumptions.

1. A neuron without lateral connections is excited by external stimuli. The times between the arrival of two successive stimuli are assumed to be independently and identically distributed with distribution function *F*_*Z*_(·).
2. Each of the input impulses has a random lifetime *τ* during which time it is stored in the memory.
3. The lifetime *τ* is a random variable with distribution function *G*_*τ*_(·). It can be noted that the case of deterministic lifetimes discussed in [3] can be obtained by setting *G*(*t*) = *H*(*t* − *τ*), where *H*(·) is the Heaviside unit function.
4. The lifetime *τ* and the time between stimuli are independent of each other.
5. When the number of stored inputs reaches a threshold value *K*, the neuron fires and the number of stored inputs in the memory are reset to one input which has a lifetime of *τ*. In this sequel, we restrict our analysis to the case *K* = 2, although the governing equations can be set up for the general case. But an analytical solution seems intractable.

In order to derive the probability density function of *T*, the interspike interval, we note that, during the interval [0, *T*], a random number *N* of impulses can occur of which the last and penultimate impulses are separated by an interval of length *τ*. Thus, the interval *T* is comprised of the sum of a random number of intervals, each of which is greater than *τ* and one last interval whose duration is less than *τ*. We first define a sequence of independent and identically distributed random variables *X*_*i*_, *i* = 1,2,…, which are distributed as *Z* but conditioned on *Z* > *τ*. Similarly, we define a conditioned random variable *Y*_*N*_ distributed as *Z* but conditioned on *Z* ≤ *τ*. Thus, *T* can be expressed as

(1) $$\begin{array}{c} T=\sum_{i=1}^{N-1}X_{i}+Y_{N}. \end{array}$$

The value *N* − 1 in (1) is a random variable representing the number of impulses which do not contribute to the firing in one spike interval. It is immediately seen that *N* follows the geometric distribution given by

(2) $$\begin{array}{c} P\left(N=n\right)=qp^{n},\;n=0,1,2,\ldots , \end{array}$$

where *q* = *P*(*Z* ≤ *τ*) and *p* = 1 − *q*.

We define the conditional distributions of *X*_*i*_ and *Y*_*N*_ as

(3) $$\begin{array}{c} \alpha \left(t\right)=P\left(t<Z<t+dt\;|\;Z>\tau \right)=\frac{f_{Z}\left(t\right)G_{\tau }\left(t\right)}{P\left(Z>\tau \right)},\\ \beta \left(t\right)=P\left(t<Z<t+dt\;|\;Z\leq \tau \right)=\frac{f_{Z}\left(t\right)\bar{G_{\tau }}\left(t\right)}{P\left(Z\leq \tau \right)}. \end{array}$$

Now,

(4) hT(t)=P(t<T<t+dt)=∑n=1∞P(t<T<t+dt ∣ N=n)P(N=n)=∑n=1∞(α(n−1)∗β)P(Z≤τ)[P(Z>τ)]n−1,

where *α* ^(*n*−1)^∗*β* is the convolution of the (*n* − 1)-fold convolution of *α*(*t*) and *β*(*t*).

Taking the Laplace transform on both sides of (4) yields

(5) $$\begin{array}{c} L_{h}\left(s\right)=\frac{L_{f\bar{G}}\left(s\right)}{1-L_{fG}\left(s\right)}, \end{array}$$

where $L_{f\bar{G}}(s)$ and *L*_*fG*_(*s*) are the Laplace transforms of the functions $f_{Z}(t)\bar{G_{\tau }}(t)$ and *f*_*Z*_(*t*)*G*_*τ*_(*t*), respectively. Given the specifications of the distributions *F* and *G*, one might be able to invert (5) to obtain the probability density function *h*(*t*). In cases where a closed form inversion of *L*_*h*_(*s*) is not possible, one can use the algorithms proposed by Abate and Whitt [23] for numerically inverting Laplace transforms which are designed especially for probability density functions.

One of the statistical characteristics in neuronal studies is the mean time between neural spikes which is used in constructing interval histograms. In our model, *E*(*T*), the mean time between two firings, is obtained by differentiating *L*_*h*_(*s*) with respect to *s* and setting *s* = 0, so that

(6) $$\begin{array}{c} E\left(T\right)=\frac{E\left(Z\right)}{P\left(Z\leq D\right)}. \end{array}$$

Also differentiating twice and setting *s* = 0 yield

(7) Var⁡(T)=E(Z2)P(Z≤D) +2E(Z)E(Z ∣ Z>D)P(Z>D)−E2(Z)P(Z≤D)2.

Hereafter, we will consider the lifetime *τ* to be a constant to illustrate our model. Let us first assume that the stimuli arrive according to exponential density *f*(*t*) = *λe* ^−*λt*^ and

(8) $$\begin{array}{c} G_{\tau }\left(t\right)=\{\begin{array}{cc} 0, & 0\leq t<\tau ,\\ 1, & t\geq \tau . \end{array} \end{array}$$

Using (5) and after some computation, we get the Laplace transform of the probability density function of interspike interval *T* as

(9) Lh(s)=λs+λ1−e−(s+λ)τ1−(λ/(s+λ))e−(s+λ)τ=∑n=1∞(λs+λ)n[e−(s+λ)(n−1)τ−e−(s+λ)nτ].

Inverting the above Laplace transform, we get density function of *T* as

(10) h(t)=λe−λt∑j=1∞λ(j−1)(j−1)!    ×[(t−(j−1)τ)(n−1)U(n−1)τ      −(t−jτ)(n−1)Unτ],

where *U*_*c*_(*t*) is the Heaviside unit step function. It should be noted that the series given above is a finite series terminating with *n* = [*T*/*τ*]. *E*(*T*) is obtained from (6) as

(11) $$\begin{array}{c} E\left(T\right)=\frac{1}{\lambda \left(1-e^{-\lambda \tau }\right)}. \end{array}$$

The variance and coefficient of variation of *T* are given by

(12) $$\begin{array}{c} \mathrm{Var}\left(T\right)=\frac{1+2\lambda \tau e^{-\lambda \tau }}{\lambda^{2}{\left(1-e^{-\lambda \tau }\right)}^{2}},\\ \text{CV}\left(T\right)=\sqrt{1+2\lambda \tau e^{-\lambda \tau }}. \end{array}$$

These results coincide with those of Vidybida [3] (see (7), (9), and (10) of his paper).

As a second example, if the stimuli arrival distribution is uniform so that

(13) $$\begin{array}{c} f\left(t\right)=\frac{1}{b-a},\;a<t<b \end{array}$$

and with constant lifetime *τ*, we obtain

(14) $$\begin{array}{c} L_{h}\left(s\right)=\frac{e^{-sa}-e^{-s\tau }}{s\left(b-a\right)-e^{-s\tau }+e^{-sb}},\\ E\left(T\right)=\frac{b^{2}-a^{2}}{2\left(\tau -a\right)}. \end{array}$$

The coefficient of variation of *T* is given by

(15) $$\begin{array}{c} \text{CV}\left(T\right)=\sqrt{\frac{2\mu_{2}\left(\tau -a\right)+\mu_{1}\left(b^{2}-2\tau^{2}+a^{2}\right)}{\mu_{1}\left(b^{2}-a^{2}\right)}}, \end{array}$$

where *μ*_1_ and *μ*_2_ are the first and second moments of *f*(*t*).

In the final example, we assume that the lifetime of the stimuli *τ* is a random variable with exponential distribution *g*(*t*) = *μe* ^−*μt*^ and the stimuli arrival distribution is also exponentially distributed with density function *f*(*t*) = *λe* ^−*λt*^ for *t* > 0. Then, from (5), we have

(16) Lh(s)=λ(s+λ)s2+s(2λ+μ)+λ2=λ(s+λ)(s−r1)(s−r2).

Inverting the above equation, we get the density function of *T* as

(17) $$\begin{array}{c} h\left(t\right)=\frac{\lambda }{r_{1}-r_{2}}\left[\left(\lambda +r_{1}\right)e^{r_{1}t}-\left(\lambda +r_{2}\right)e^{r_{2}t}\right], \end{array}$$

where *r*_1_ and *r*_2_ are the real roots of the equation *s* ^2^ + *s*(2*λ* + *μ*) + *λ* ^2^ = 0. The mean and coefficient of variation of *T* are given by

(18) $$\begin{array}{c} E\left(T\right)=\frac{\lambda +\mu }{\lambda },\\ \text{CV}\left(T\right)=\sqrt{\frac{\lambda +4\lambda \mu -\mu }{\lambda +\mu }}. \end{array}$$

An important property of the interspike intervals in the present model that will prove useful in our analysis, which can be intuitively verified, is as follows.

*Assertion.* If *T* ^*τ*^ is the random variable of the firing intervals, based on the lifetime *τ*, then *T* ^*τ*_1_^ > *T* ^*τ*_2_^, where *τ*_1_ < *τ*_2_, in the stochastic ordering sense. The above assertion implies that for decreasing lifetime *τ*, the probability of firing for a fixed *t* is increasing. Also as *τ* → *∞*, every stimuli gives rise to a firing while as *τ* → 0, the neuron cannot fire.

## 3. Perspectives on the Passage from a Single Binding Neuron to a Network

In order to study the role of inhibitory and excitatory connections amongst neurons with a mathematically tractable model, Cottrell et al. [6] introduced the hourglass model. The main idea behind the hourglass model [17, 24] is to associate, at each time *t*, the expected time *X*(*t*) that remains for the neuron to fire. This is to be contrasted with the traditional models, where the membrane potential level *V*(*t*) is the variable of interest. If *U* denotes the random variable denoting the interspike interval, the stochastic process {*X*(*t*), *t* ≥ 0} satisfies the spike train as follows:

(19) $$\begin{array}{c} X\left(t+dt\right)=X\left(t\right)-dt\;\text{if}\;X\left(t\right)>0,\\ X\left(t+dt\right)=U-dt\;\text{if}\;X\left(t\right)=0. \end{array}$$

{*X*(*t*), *t* ≥ 0} is Markov process which decays linearly with slope −1 in between firings. The model is called hourglass model because one can visualize an hourglass being refilled with an amount *U* after each firing. With our success in finding the expected time to fire for the binding neurons, the above model is completely specified. The above-said hourglass model for a single neuron can be extended to a network of connected binding neurons along the lines of Cottrell et al. [6].

Consider a network of *n* binding neurons which are interconnected. Each of the neurons in the network has its own spiking activity due to external stimulations, as modeled in Section 2. Consider a typical neuron *i* in the network. Let us denote by *I*_*i*_ and *E*_*i*_, respectively, the set of neurons in the network which are inhibited and excited by neuron *i*. We assume that when each time neuron *i* fires, it triggers a short input to all its connected neighboring neurons. In the case of inhibitory connections, the effect of such an input is to reset the expected firing time for every neuron in *I*_*i*_. Note that this results in an increase *w*_*ij*_ in the expected firing time of all neurons *j* in the set *I*_*i*_. Similarly, in the case of excitatory connections, the effect of such an input is to reset the expected firing time of every neuron in *E*_*i*_, which leads to a decrease *w*_*ij*_ in the expected firing times of all neurons *j* in the set *E*_*i*_. Thus, the positive or negative delays *w*_*ij*_ in the sets *I*_*i*_ and *E*_*i*_ are functions of the lifetime *τ* of the neurons.

Following Cottrell et al. [6], the behavior of the network can now be written for every neuron *i* in the network as follows. Let *X*_*i*_(*t*) be the remaining expected time to fire of the neuron *i* in the network at time *t* and let *U*_*i*_ be the expected time to fire. If *w*_*ij*_ is the amount of increase (or decrease) in the interconnected inhibitory (excitatory) neuron *j* in the network, then we have the following.

If *X*_*i*_(*t*) ≠ 0,

(20) $$\begin{array}{c} X_{i}\left(t+dt\right)=X_{i}\left(t\right)-dt;\;\forall i. \end{array}$$

If *X*_*i*_(*t*) = 0,

(21) Xj(t+dt)={Ui−dt,for    j=i,Xj(t)+wij−dt,if  j  ∈  Ii,max⁡{0,Xj(t)−wij−dt},if  j  ∈  Ei.

It can be shown that the process {*X*_*i*_(*t*), *i* = 1,2,…*n*} is an irreducible aperiodic Markov chain. To visualize the behavior of this Markov chain, we construct a discrete-event simulation model of a neural network composed of one hundred neurons organized in a grid of 10 × 10. Each neuron in the network is connected with its laterally adjacent neurons (left, right, up, and down). Following the model mentioned in Cottrell et al. [6], we build this example assuming that all the lateral connections are inhibitory.

Given the above mentioned network characteristics, the behavior of a single neuron *i* in this grid is modeled as follows. At each unit of time, the remaining time until firing *X*_*i*_(*t*) is reduced by one unit (showing the behavior of the hourglass model of a single neuron). When *X*_*i*_(*t*) reaches zero, neuron *i* fires and therefore sends stimuli to all of its laterally connected neurons. When this occurs the value of *X*_*i*_(*t*) is refilled with expected time for the neuron to fire. In our simulation studies, we use the case of constant lifetime *τ* so that the refill amount is given by (11) with *λ* = 15  second^−1^. We note that with such a formulation, the remaining time to fire of any neuron in the network is a function of the parameters *τ*, the input lifetime, and *w*_*ij*_, the increment (decrement). Each neuron of this network is simulated in a submodel following the hourglass structure. In each individual neuron submodel, the transmitters arrive at the neuron in the form of simulation model entities and reduce its remaining time to fire. A module within each submodel keeps track of the remaining time to fire and updates this value each time a transmitter arrives. Eventually, when the value of the remaining time to fire reaches zero, the neuron fires and immediately after firing, the hourglass is refilled. Once the neuron fires, the stimulus reaches the adjacent neurons. To model the connection of each neuron with the rest of the network, we build a submodel to control the connections. Upon firing a signal by each neuron, the stimulus is received by the sub model of connections. This submodel distributes the signal to the laterally connected individual neurons in the network which are governed by a similar submodel for the individual neurons. In this example, for computational purposes we take, *w* = *w*_*ij*_ to be constant for all neurons *i* and *j*; we present our results for the three cases, where we evaluate the effect of changing *τ* and *w* for values of *τ* = 1,5, 7 and *w* = 4,15,30. In order to interpret the results, we categorize the neurons within this network depending on their spike activity. If a neuron has less than 5 spikes, it is considered a silent neuron (colored white), between 6 and 200 spikes is considered a medium activity neuron (gray), and above 200 spikes is considered a high activity neuron (blue).

**Figure 1:**
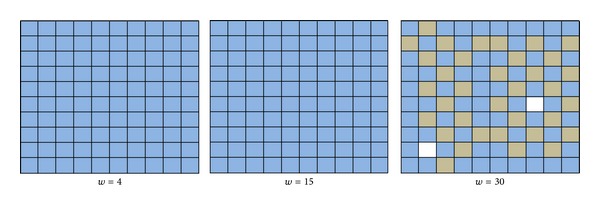
Simulation results of the 10 × 10-neuron network with *τ* = 1.

**Figure 2:**
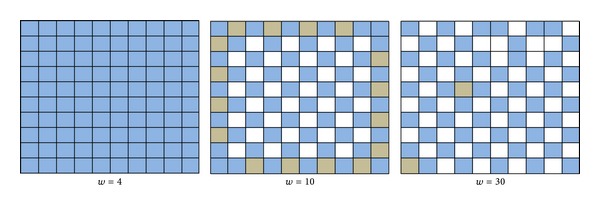
Simulation results of the 10 × 10-neuron network with *τ* = 5.

**Figure 3:**
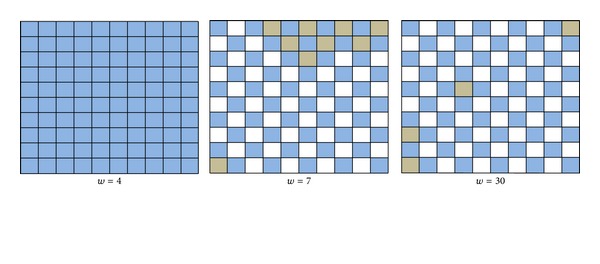
Simulation results of the 10 × 10-neuron network with *τ* = 7.

In each case (i.e., *τ* = 1,5, 7), we show results beginning with *w* = 4, where all the neurons are active. Then, we present the matrix for the first value of *w* at which we start observing silent neurons and we also present the results for *w* = 30. These results, for Case 1 (*τ* = 1), are shown in Figure 1. In this case, the neurons that constitute the network are all active until *w* takes the value of 30, where we can observe two silent neurons which are not on the matrix borders. In Figure 2, results for Case 2 are presented when *τ* = 5. In this case, at *w* = 10, the neurons start becoming inactive. In this case, the number of silent neurons is 32, all located in the middle part of the matrix and their adjacent neurons are all active. However, the level of activity decreases on the borders. When we increase the value of *w* to 30, some silent neurons start appearing on the borders of the matrix. In Figure 3, the results of Case 3 with *τ* = 7 are presented. In this case, the value of *w* at which neurons become inactive is lower than that of the two previous cases which occurs at *w* = 7. Also, the number of silent neurons increases at a higher rate than that of the last two cases as we observe silent neurons in the matrix border even at *w* = 7. Results similar to Case 3 were observed in all cases of *τ* with higher values which means that by incrementing the value of *w*, more silent neurons appear in the network. The early neurons that become inactive are the ones in the middle of the matrix and as the value of *w* increases, border neurons also start to become silent. Comparing Figures 1, 2, and 3 also reveals that as we increase the value of *τ* and keep the value of *w* constant (as seen in all instances with *w* = 30), the number of silent neurons grows. This means that *τ* and *w* are proportional in terms of the number of silent neurons. These observations, although not conclusive, are consistent with the example presented in [6], where increasing the value of *w* decreases the activity of the network, and this inactivity starts appearing in the middle of the matrix first, and as *w* increases, it propagates to the borders as well.

Finally, let us compare our approach with that of Vidybida [3] and Cottrell et al. [6] whose works have been the basis of the present work. Vidybida's model moves away from the existing models of physiochemical quantities towards models operating in terms of input impulses and their lifetimes. This approach encompasses the LIF models. We seek to generalize his approach taking the model neurons more towards biological neurons. The renewal process of assumptions of the input impulses is a step in that direction, although we still are in the regime of independence of input events. In our view, a realistic neuron model should take into account the autocorrelational structures of the input. Another element of realism is the randomness of the lifetimes of the input events. In real neurons, electrochemical transience supports deterministic lifetime of impulses, however, in mass service theory the service time which is the counterpart of lifetime *τ* is random. Further, the deterministic *τ* can easily be obtained as a particular case of random *τ*. The present approach otherwise retains all the other ingredients of Vidybida's approach.

Existing network models operate from two ends; at one end, they use physiochemical properties like ion conduction, propagation of signals through axons, and so forth. At the other end there are models dealing with firing rates and histograms only. However, Cottrell's hourglass model takes a middle path in which the variable of interest is the remaining time to fire for the neuron. Armed with probability distribution and mean time to firing of individual binding neurons, our approach integrates Vidybida's model neuron and Cottrell's hourglass model. As contrasted with Cottrell's model in which the activity of neurons in the network is based on the input *w*_*ij*_ only, our approach uses both *w*_*ij*_ as well as *τ*, the lifetime of the input impulses as factors contributing to the network activity. Our limited simulation study conforms to the results of Cottrell that the increasing values *w*_*ij*_ decrease the neuronal activity and this inactivity starts to appear in the centre of the network and propagates to the boundaries as well with the increasing *w*_*ij*_. However, an in-depth simulation study is needed to understand neuronal activity. In this regard, we wish to mention that *w*_*ij*_ could be made to depend on the Euclidean distance between neurons *i* and *j* in the network.

## 4. Conclusion

This paper generalizes the binding neuron model of Vidybida in several aspects. The input stimuli are governed by a renewal process which gives the modeler flexibility in modeling. The lifetime of the inputs which depends on the location of the neuron under consideration is naturally assumed to be random variables. A noteworthy aspect is the explicit analytical expressions for the probability distribution and statistical characteristics of the time to firing of the neuron. This will be very useful in the study of neuronal networks. The paper also attempts to carry forward the modeling from single binding neuron to a network of such neurons using the hourglass model.
